# Warming and predation risk only weakly shape size-mediated priority effects in a cannibalistic damselfly

**DOI:** 10.1038/s41598-022-22110-6

**Published:** 2022-10-15

**Authors:** Mateusz Raczyński, Robby Stoks, Szymon Sniegula

**Affiliations:** 1 Department of Ecosystem Conservation, Institute of Nature Conservation, Polish Academy of Sciences, al. Adama Mickiewicza 33, 31-120 Krakow, Poland; 2 Evolutionary Stress Ecology and Ecotoxicology, University of Leuven, Leuven, Belgium

**Keywords:** Physiology, Environmental sciences, Zoology, Entomology, Ecology, Climate-change ecology, Freshwater ecology, Climate sciences, Climate change, Ecology, Climate-change ecology, Freshwater ecology

## Abstract

Differences in hatching dates can shape intraspecific interactions through size-mediated priority effects (SMPE), a phenomenon where bigger, early hatched individuals gain advantage over smaller, late hatched ones. However, it remains unclear to what extent and how SMPE are affected by key environmental factors such as warming and predation risk imposed by top predators. We studied effects of warming (low and high temperature) and predation risk (presence and absence of predator cues of perch) on SMPE in life history and physiological traits in the cannibalistic damselfly *Ischnura elegans*. We induced SMPE in the laboratory by manipulating hatching dates, creating following groups: early and late hatchlings reared in separate containers, and mixed phenology groups where early and late hatchlings shared the same containers. We found strong SMPE for survival and emergence success, with the highest values in early larvae of mixed phenology groups and the lowest values in late larvae of mixed phenology groups. Neither temperature nor predator cues affected SMPE for these two traits. The other life history traits (development rate and mass at emergence) did not show SMPE, but were affected by temperature and predator cues. A tendency for SMPE was found for protein content, in the high temperature treatment. The other physiological traits (phenoloxidase activity and fat content) showed fixed expressions across treatments, indicating decoupling between physiology and life history. The results underline that SMPEs are trait-dependent, and only weakly or not affected by temperature and predation risk.

## Introduction

One of the biggest challenges in ecology is to understand and predict the impact of climate change on species and populations^1,2^. However, such understanding is complicated by the fact that species are embedded in complex communities. Therefore, it is not enough to understand how species are affected by warming per se, but also how warming changes their antagonistic^3,4^ and synergistic^5,6^ interactions. Changes in temperature have indeed been shown to affect antagonistic interactions between organisms^7–10^. These changes in interactions can be caused by shifts in phenological events^11^, for example, by changes in relative hatching dates among interactive organisms^12,13^. In predator–prey systems, higher temperatures may lead to increased activity and encounter rates that benefits predators in terms of higher food acquisition, earlier time at maturity and larger final size^14,15^ through changes in physiology^16,17^.

Specifically for cannibalistic interactions, the outcome of intraspecific encounters is strongly determined by the difference in body size^18–20^, and encounter rates and cannibalism rates increase under warming^21,22^. In such situations, larger individuals take advantage over smaller individuals leading to so called size-mediated priority effects, SMPE^14^. It has been shown that individuals that appear early in a habitat show SMPE in life history (e.g. increased adult mass and survival) and in physiological traits (e.g. increased metabolic rates and improved immune function)^23–25^. There is accumulating evidence that changes in phenological events such as relative hatching dates increase the magnitude of SMPE due to enlarged variation in relative body size of interacting animals^9,26^. Although the theoretical backgrounds of priority effects in a warming world have been explored^4,27^, to our knowledge there have been few empirical approaches that linked life history with physiology in the context of SMPE caused by temperature-mediated hatching dates^28,29^.

Predator–prey interactions can have direct consumptive, and indirect non-consumptive effects. Non-consumptive predator effects refer to reductions in prey fitness through behavioural and physiological changes^30–33^. The direction and intensity of non-consumptive predator effects may vary depending on the characteristics of the predator–prey couple, for example the predator:prey body size ratio which determines predator gape limitation^34^. Non-consumptive predator effects caused by visual or chemical predator cues can lead to reduced prey activity, food intake and growth^35–38^. Instead, prey may also increase growth rate to escape gape-limited predators^39^, but this often leads to costs in terms of a decreased size at maturity^40^ and a reduced ability to neutralize free radicals^41,42^. Non-consumptive predator effects can have equally or even more negative consequences for prey communities than consumptive effects^43,44^. However, it is still unclear whether and how the non-consumptive predator effects impact SMPE in prey, especially when prey represent intermediate, cannibalistic predators in a food chain, and the predators are at the top of the food chain. Furthermore, the presence of predator cues may change the effect of warming on prey life history, e.g., by reducing the growth rate in prey^45,46^. This makes the interaction of temperature and predator cues especially important in predicting the final outcome of the predator–prey interactions, hence also of SMPE.

Here, we studied combined consumptive (intraspecific SMPE) and non-consumptive (cues of perch, a top predator in ponds) predator effects on life history and physiology in the cannibalistic damselfly *Ischnura elegans* (an intermediate predator in ponds). By crossing consumptive and non-consumptive predator effects with two thermal conditions simulating the current and the predicted increased temperature by 2100 we could assess how both predator-induced effects may change under future warming. We examined SMPE in larval and adult life history and physiological traits and whether non-consumptive top predator effects in combination with increased temperature experienced during damselfly egg and larval stages affect SMPE. We had following set of hypotheses. (1) *I. elegans* shows SMPE in life history and physiological traits. We expected early hatchlings to have advantage over late hatchlings in terms of food acquisition, including cannibalism, leading early hatchlings to show higher values for life history traits (survival, development rate and mass at emergence) and physiological traits (increased energy storage in fat and proteins, and an increased investment in immune function measured as phenoloxidase activity)^4,9,47–50^. (2) SMPE is more pronounced or more likely at the higher temperature as this leads to increased food acquisition through increased activity^51–54^, and more/earlier cannibalism^22^, but see^55^. (3) SMPE in life history and physiological traits is less pronounced when larvae experience additional stress imposed by top predator cues. *Ischnura* species show reduced activity and metabolic rate in the presence of fish predator cues^56,57^, which in turn might cause reduced food acquisition, including cannibalism, and decreased intensity of SMPE^58,59^.

## Materials and methods

### Study species and collection

In this experiment we used the damselfly *I. elegans* as focal species. As top predator, we used the European perch (*Perca fluviatilis*) to impose non-consumptive predator effects on the damselfly. *I. elegans* a common insect species in Europe, occurring from northern Spain to central Sweden^60^. Central Europe populations are uni- and bivoltine (one or two generations per year, respectively), depending on the thermal conditions^61^. Larvae hatch 2–3 weeks after egg laying. Eggs and larval stages commonly share habitats with predatory fish^62^. Fish cues can affect egg and larval life histories and physiology in the study species^63,64^.

Adult *I. elegans* females were collected at a pond in Zabierzów Bocheński, Poland (50°03′16.3"N, 20°19′45.7"E). This fish pond contains *P. fluviatilis*. In total, 40 and 36 females were caught in copula on 22 June 2019 (i.e., early group) and on 7 July 2019 (i.e., late group). Females were individually placed in plastic cups with perforated lids and wet filter paper for egg laying, and transported by car in a Styrofoam box to the Institute of Nature Conservation PAS (INC PAS), Krakow, Poland. Adult females were kept in a room at a temperature of 24 °C and natural daylight (photoperiod). Females laid eggs within three days after they had been field-collected. In total 22 clutches were used for the early group treatment, and 26 clutches for the late group treatment. After egg laying, females were released in their natural population.

Ten *P. fluviatilis* (age: 1+) were caught in Dobczyce lake (49°52′27″N, 20°2′55″E) on 19 June 2019. Five fish were used in the experiment, another five were used as a backup. Fish collection and housing were done with a permission from the Local Ethical Committee (ref. 261/2019). Fish were fed frozen *Chironomidae* larvae daily. Fish were not fed with live damselfly larvae, and this to eliminate alarm cues released by larvae exposed to predation^65^.

### Housing

**Figure 1:**
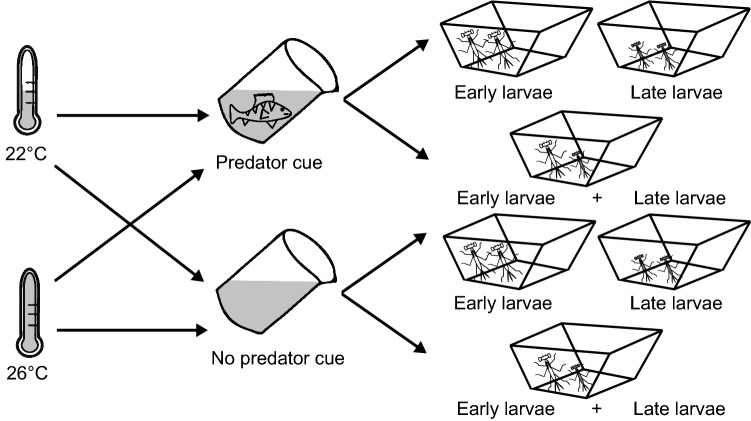
A schematic graph visualizing the full factorial experimental design with two temperature treatment groups crossed with two different predator cue treatment groups, that were each divided into four different phenology groups, resulting in 16 treatment combination groups. Note that in reality 16 larvae were present per container.

Egg clutches from early collected females were pooled, and the same was done with eggs from late collected females. The two hatching phenology groups, early (E) and late (L), had 16 days difference in hatching dates, corresponding to the time interval between adult female field collection dates. Such difference in hatching dates occurs in the natural populations because of the long *I. elegans* mating season and mixed voltinism in the sampling region^61,66^. We also created mixed phenology groups, where early hatched individuals shared the same container with late hatched individuals. Note that for the statistical analyses early and late hatched individuals in mixed phenology groups were considered as two different groups, E + L and L + E, where the group E + L referred to the early larvae in the presence of late larvae, and the group L + E to the late larvae in the presence of early larvae. This resulted in four phenology groups: non-mixed E and L, and mixed E + L and L + E. In the non-mixed phenology groups sets of 16 larvae of the same phenology group (E or L) were placed in separate containers, and in mixed phenology groups 8 larvae from E and 8 larvae from L phenology groups were placed in the same container, creating E + L and L + E phenology groups. This way all containers contained 16 larvae. This represents potential scenario for priority effects occurring in nature when comparing densities of unsynchronized hatchlings, compared to otherwise synchronised groups^27^. Each phenology group was studied under the four combinations of two top predator treatments (fish predator cues present and absent) and two temperature treatments (22 °C and 26 °C, hereafter, low and high temperature). Therefore, the set of pooled clutches of each phenology group were separated in four subsets, each subset being assigned to one predator-by-temperature treatment combination. This was done before hatching by cutting paper filters on which eggs had been laid and transferring these to separate containers. The temperature treatment started at hatching. The low temperature treatment was based on average temperatures in shallow ponds^67^, while the high temperature treatment matched the predicted mean temperature increase by 2100 under IPCC scenario RCP 8.5^68^. This created the following full factorial crossed design: 3 phenology groups × 2 predator cue treatments × 2 temperature treatments × 12 replicated containers × 16 larvae = 2304 individuals at the start (Fig. 1). Throughout the experiment we used a constant photoperiod of L:D 16:8 h, which corresponds to the summer photoperiod, i.e., peak of the larval growth season, at the collection site. We used two climate incubators (Pol-Eko ST 700) for damselfly rearing.

Hatching took place on 6 July 2020 (E group) and 22 July 2020 (L group) at the high temperature, and on 11 July 2020 (E group) and 27 July 2020 (L group) at the low temperature treatment. At hatching, we randomly chose 16 larvae from E and L groups and transferred them to separate containers (16 × 12 cm, height 8 cm) filled with 600 mL of dechlorinated tap water and two nylon net strips, providing hiding space for larvae and climbing structure during emergence. In E + L and L + E groups, we randomly choose 8 larvae from the E group and 16 days later added 8 larvae from the L group. Larvae were fed twice a day (morning and afternoon feeding) with *Artemia salina* nauplii. During the feeding, E and L groups received 10 portions/container (mean = 201.9 nauplii/portion, SD = 17.2). In mixed groups, early hatched larvae received five portions until late hatched larvae were introduced to the same containers. From this time, mixed phenology groups received 10 portions/container.

Every other day, 150 mL of water in every container was refilled with water containing predator cues or no predator cues. Earlier studies have shown that chemical cues of aquatic predators have an average half-life degradation time of ca. 36.5 h^69^. Previous experiments on non-consumptive predator effects in damselfly larvae supported this^33,64^.

To distinguish early from late hatched individuals in E + L and L + E groups, we cut the tibia of either one left or one right middle leg. Individuals from E and L groups were marked the same way. The larvae were marked when 30 days old. At that moment, individuals from the two hatching phenology groups could be easily distinguished by size. A preliminary study showed that a 15–20 days difference in hatching dates is sufficient for *I. elegans* larvae to complete two moults regardless of temperature and predator cue treatment (unpublished data). This marking persists until emergence and does not impact the measured traits^9,70^.

Freshly emerged individuals were individually transferred to a dry plastic cup and kept for 24 h until the cuticle hardened. Next, damselflies were weighted and frozen at −80 °C for physiology analyses. The experiment ended when the last damselfly larvae emerged.

### Response variables

#### Life history

The survival was noted daily between hatching and emergence. Individuals that emerged with fully developed body and wing parts were considered to have emerged successfully. Temperate damselflies are highly cannibalistic and cannibalism increases with increasing body size differences^71^. Based on previous studies on *I. elegans* with larvae reared in groups^25^ or individually^64^ and on another damselfly species, *Lestes sponsa*, where intrinsic mortality versus cannibalism was determined^29^, we assumed that intrinsic mortality was less likely to occur than mortality caused by cannibalism. Larval development time was measured as the number of days between hatching and emergence. One day after emergence, damselfly wet mass was measured to the nearest 0.1 mg with the use of an electronic balance (Radwag AS.62). The growth rate was calculated as adult wet mass divided by the number of days between hatching and emergence. Here, we did not correct for the hatchling mass because we assumed that hatchling mass did not affect adult mass, as shown in^72^. Also, early handling of hatchlings might interfere with results and larval survival in odonates^73^.

#### Physiology

For physiological analyses, damselfly bodies without legs and wings were grinded with phosphate buffer solution (15 µL for each milligram of wet mass) and centrifuged at 10,000 g for 5 min at 4 °C. All physiology analyses were done on homogenates.

The classical procedure for measuring total body fat in insects^74^ was optimized for damselfly bodies. A volume of 8 µL homogenate was mixed with 56 µL 100% sulfuric acid, and heated for 20 min at 150 °C. After cooling down, 64 µL Milli-Q-Water was added. Of this mixture, 30 µL was put in a well of a 384-well microliter plate, and absorbance was measured at 340 nm. The measurements were made on an Infinite M2000 (TECAN) plate reader. To convert absorbances into fat contents, the standard curve of glyceryl tripalmitate was used. The average of three technical replicates per sample was used in the statistical analyses.

Protein content (µg of protein/mg of body mass) was determined using the Bradford^75^ method. Of the homogenate, 1µL was mixed with 160 µL of Milli-Q-Water and 40 µL of Bio-Rad Protein Dye. After five minutes of incubation at 25 °C, the absorbance was measured at 595 nm and converted into protein contents using standard curves of bovine serum albumin. The measurements were repeated three times per sample, and the average values used for statistical analyses.

A modified version of the assay described in^76^ was used for determining PO activity. Of the homogenate, 10 µL was mixed with 10 µL of phosphoric buffered saline and 5 µL of chymotrypsin. The mixture was put in wells of a 384-well microtiter plate. Afterwards, the samples were incubated for 5 min at room temperature. After incubation, the substrate L-DOPA (1.966 mg dihydroxyphenyl-L-alanine per 1 mL of PBS-buffer) was added and mixed with the samples. Immediately afterwards, the linear increase in absorbance was measured at 490 nm every 20 s for 30 min at 30 °C. The PO activity was quantified as the slope of the reaction curve, and the average of two technical replicates was used for statistical analyses.

#### Statistical methods

All analyses were run using R 4.0.4^77^. Generalized mixed models with a binomial distribution were used to separately analyse the survival and emergence success (*glmer* function in the lme4 package^78^). The other life history traits (development time, wet mass and growth rate) and the physiological traits (PO activity, fat and protein contents) were analysed using linear mixed models (*lmer* function in the lme4 package^78^). In all models, phenology group, top predator cue treatment, temperature treatment and sex were entered as fixed effects. Initially, models with all possible interactions were run. Interaction terms with *p* > 0.1 were removed from the final models. In all models, container nested within phenology groups were used as random variable. SMPE would be indicated by the pattern where the trait value in the E + L group would be statistically higher (survival, mass, growth rate, fat content, protein content and PO activity) or lower (development time) than in the other phenology groups, and this because of a competitive advantage of early hatched individuals over late hatched ones within mixed-phenology group. If a factor with more than two levels or any interaction term was found statistically significant, post hoc Tukey HSD tests (function *lsmeans*) were run to test pairwise between-level differences. Because of low number of surviving larvae in the L + E group, this group was excluded from all analyses, except for survival until emergence and emergence success.

### Ethics declaration

All animal experiments were approved by First Local Ethical Committee for Animal Experiments in Krakow, Poland, and conducted according to Committee guidelines and regulations, reference number 261/2019.

### ARRIVE declaration

Manuscript confirming our study has been reported in accordance with ARRIVE guidelines.

## Results

### Life history

**Figure 2:**
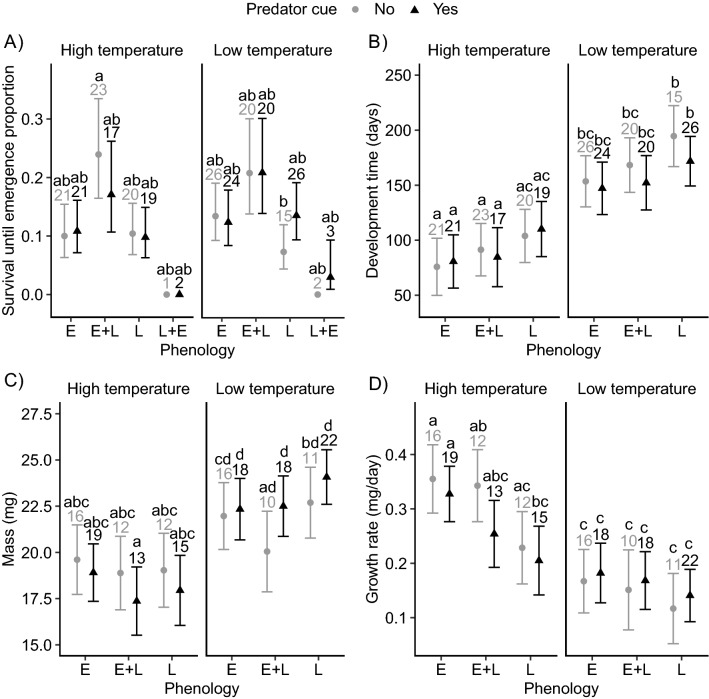
(**A**) Proportion of survival, (**B**) development time, (**C**) adult mass and (**D**) growth rate across different phenology groups (E, E + L, L and L + E), temperatures (high and low) and top predator cues (no and yes). Error bars indicate estimated 95% CI. The numbers on top of error bars represent the number of larvae within each group. E = early larvae, E + L = early larvae in mixed phenology group, L = late larvae, L + E = late larvae in mixed phenology group. All phenological groups are presented, but groups with N < 2 have error bars removed from the plots for clarity. Because of low sample sizes in the L + E phenology group, the L + E group was removed from all analyses, except for analysis on survival rate and emergence success. Letter codes indicate significant differences between phenology groups across both temperatures and predator cue treatments.

There was a SMPE in survival until emergence, with other factors (temperature, predator cues and sex) not affecting the magnitude of SMPE in this trait. Phenology affected survival until emergence (Fig. 2A, Table 1), with larvae from the E + L group showing the highest survival, and larvae from the L + E group showing the lowest survival (Fig. 2A). None of the interaction terms were significant (Fig. 2A, Table 1). The average percentage of survival in E + L, E, L and L + E groups were 20.1%, 11.98%, 10.29% and 2.1%, respectively (percentages based on raw data). The pattern in SMPE in emergence success was the same as for survival until emergence (Fig. S1, Table 1). There was no SMPE pattern in the other life history traits (development time, adult mass and growth rate).

**Table 1:** Results from mixed models on life history and physiological traits.

Predictor	Df	χ^2^	*p*
Survival until emergence			
Phenology	3	55.59	** < 0.001**
Temperature	1	0.54	0.46
Sex	1	0.04	0.84
Predator cues	1	0.11	0.73
Emergence success			
Phenology	3	46.95	** < 0.001**
Temperature	1	0.17	0.68
Sex	1	0.08	0.78
Predator cues	1	0.47	0.49
Development time			
Phenology	2	11.97	**0.003**
Temperature	1	110.24	** < 0.001**
Sex	1	2.14	0.14
Predator cues	1	0.64	0.42
Mass at emergence			
Phenology	2	3.73	0.15
Temperature	1	53.82	** < 0.001**
Sex	1	16.26	** < 0.001**
Predator cues	1	0.01	0.91
Temperature × predator cues	2	7.75	**0.02**
Growth rate			
Phenology	2	22.79	** < 0.001**
Temperature	1	63.1	** < 0.001**
Sex	1	0.72	0.4
Predator cues	1	0.78	0.38
Temperature × predator cues	2	5.12	0.08
Phenoloxidase activity			
Phenology	2	1.094	0.579
Temperature	1	1.434	0.231
Sex	1	0.01	0.922
Predator cues	1	0.913	0.339
Fat content			
Phenology	2	0.093	0.955
Temperature	1	0.796	0.231
Sex	1	0.244	0.621
Predator cues	1	2.302	0.373
Protein content			
Phenology	2	1.444	0.486
Temperature	1	0.796	0.654
Sex	1	0.786	0.375
Predator cues	1	0.097	0.755
Phenology × temperature	2	5.221	0.074
Phenology × sex	2	4.943	0.084

Final models included all fixed effects and interactions with *p*-values < 0.1, whereby *p*-values  ≤ 0.05 were considered significant. Except for the analysis of survival until emergence and emergence success, the L + E phenology group was excluded from analyses due to low sample size (N ≤ 2).

The phenology treatment had a significant effect on development time (Table 1), with larvae from E and E + L groups taking shorter time for development than larvae from the L group (Fig. 2B). Development time was shorter at the high than at the low temperature (Fig. 2B). Predator cues and sex had no effect on development time (Fig. 2B. Table 1).

The phenology treatment did not affect adult mass (Fig. 2C, Table 1). Larvae reared at the low temperature emerged at a higher mass than larvae reared at the high temperature (Fig. 2C, Table 1). Predator cues increased the temperature effect on mass, further decreasing mass in high temperature and increasing mass in low temperature (temperature × predator cue interaction, Fig. 2 and Fig. S2). Females had a higher mass than males (Tables 1, Fig. S3).

The phenology affected the growth rate (Fig. 2D, Table 1). Larvae from E and E + L groups had higher growth rates than larvae from the L group (Fig. 2D, Tables 1). Larvae grew faster at the high temperature (Fig. 2D, Tables 1). Predator cues tended to decrease the effect of temperature on growth rate (temperature × predator cue interaction, P = 0.08, Fig. 2D, Fig. S4, Table 1). Sexes did not differ in growth rate (Table 1).

### Physiological traits

**Figure 3:**
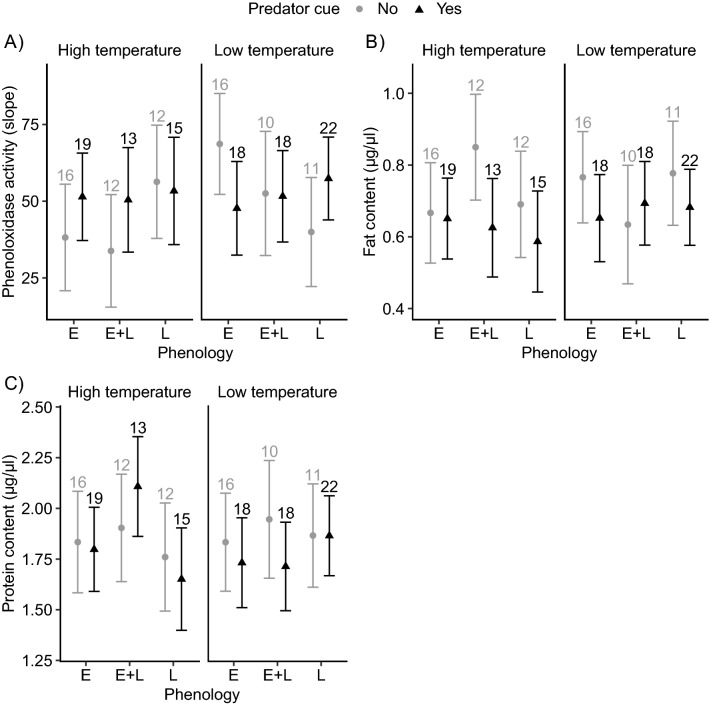
(**A**) Phenoloxidase activity, (**B**) fat content and (**C**) protein content, across different phenology groups (E, E + L and L), temperatures (high and low) and predator cues (no and yes). Error bars indicate estimated 95% CI. The numbers on top of error bars represent the number of larvae within each group. Letter codes were not added due to lack of support of statistically significant differences between groups from post-hoc tests. Abbreviations as in Fig. 2.

There were no SMPE patterns in phenoloxidase (PO) activity and fat content, and a trend for SMPE in protein content. None of the factors affected PO activity, fat content and protein content (Fig. 3A–C, Table 1). However, two interaction terms for protein content showed a trend. The high temperature tended to increase the phenology effect, with the E + L group reared at the high temperature having the highest protein content (phenology × temperature interaction, Figs. 3C, S5, Table 1), indicating SMPE at the high temperature. Males in the L group had a higher protein content than females, while the opposite pattern was present in the E group (phenology × sex, Fig . S6, Table 1). These interaction terms were not supported by the post-hoc tests.

## Discussion

We found a SMPE for two life history traits (survival until emergence and emergence success) that was similar across both temperatures supporting the first hypothesis, but not the second hypothesis. In addition, we detected a trend for SMPE for one physiological trait (protein content) but only at the high temperature matching our second hypothesis. Expression of other life history traits were in most cases affected by warming and predator cues, but for these traits we did not find SMPEs. The other physiological traits that we quantified showed fixed expressions across treatments, indicating that life history and physiological traits were to some extent decoupled in the study system.

Consistent with SMPE patterns, survival and emergence success were highest in larvae in the E + L group and lowest in those of the L + E group. These results support previous ones^9,18,29,79,80^ and confirm that intraspecific competition, including cannibalism, benefits larger individuals. In addition, early-hatched larvae in mixed phenology groups may have benefited from an increased accessibility to food due to a reduced number of competitors in the containers, i.e. thinning effects^81^. Contrary to the second and third predictions, neither temperature nor predator cues affected the strength of SMPEs in survival and emergence success. We suggest that the impact of these two factors were offset by antagonistic larval interactions. Antagonistic interactions can change larval behavior to avoid predation^32^. Previous studies showed that life history traits in damselflies are altered by non-consumptive predator stress^33,53,64,82–84^ and temperature^53,85,86^. But in these studies, the focus was on predator stress on the egg stage or on individually reared larvae, thereby precluding cannibalism. Our current results add that SMPEs in key life history traits can affect population size, but that the strength of SMPEs is weakly altered by other environmental factors.

Despite the highest survival until successful emergence of E + L individuals, we did not detect SMPE in development rate. This result does not support previous results in other ectotherms, including the damselfly *L. sponsa*, which showed SMPE in development time as well as in other life history traits^9,12,87^. In the case of *L. sponsa*, early larvae from mixed groups had the shortest development times^9^. Therefore, we suggest that the differences could be caused by different life cycle characteristics. *L. sponsa* overwinters in the egg stage and is a strictly univoltine damselfly, while *I. elegans* overwinters in the larval stage and has a variable voltinism—with uni- and bivoltine life cycles in the study region^61,66^. These life cycle characteristics influence larval behavior, life history and physiology^88^. As species under high time constraints usually grow and develop faster^89,90^, a strictly univoltine species, *L. sponsa*, due to its short larval period after wintering in the egg stage is under higher pressure for rapid growth compared to the univoltine *I. elegans,* which spends winter in the larval stage. However, a fraction of the *I. elegans* population may complete a second generation within the season (this as a result of cohort splitting, resulting in univoltine and bivoltine fractions), hence proceeds for direct larval development and emergence with no overwintering stage, and therefore is likely more time constrained (but still less than egg-overwintering *L. sponsa*), than the larval overwintering univoltine fraction^88^. Hence, the bivoltine fraction is likely more prone for SMPE. In the experiment, all phenology groups reared at the high temperature finished their larval development and emerged within 100 days (Fig. 2B). This fits the time necessary for the bivoltine fraction to finish its second generation within a season, especially in high temperature conditions, as recorded in local populations of *I. elegans*^66,91^. More studies, preferably in (semi)natural thermo-photoperiod conditions, allowing larval direct development until emergence (bivoltine) and larval overwintering (univoltine) are needed to clarify the effect of within population variation in voltinism on SMPE in damselflies.

Early hatched *I. elegans* from both mixed- and non-mixed phenology groups had shorter development times than late hatchlings. Shorter development times in early hatchlings were accompanied with elevated growth rates, and this led early hatchlings to reach similar mass at emergence as late hatchlings. Hence, there was apparently no trade-off between age and mass at emergence, which is often reported in ectotherms^92–96^, but see^97^. These plastic life history responses of early hatchlings may be adaptive. Early emerged individuals mature early in the season and have higher mating success than delayed ones^98^. Usually there is also a positive association between adult mass and components of mating success^99,100^. Additionally, we did not detect mortality costs of fast development rate, which is often reported^101–103^. Early emergence is likely adaptive by allowing the completion of an extra generation within a year, i.e. bivoltinism, especially when temperature conditions are permissive^61,66,104^. However, selection for early emergence is probably relaxed because of highly unsynchronized mating over the flight season in *I. elegans*^105^. Contrary, in damselflies with synchronized, early season emergence and mating such as *Coenagrion armatum*^106^, *Coenagrion hastulatum* or *Coenagrion puella*^107^ selection for early date emergence, fast development and early maturation is likely strong. Finally, maternal effects could have also played a role in shaping the faster development of early vs late offspring. It has been reported that when mothers age, their condition may drop and the offspring quality decrease^108–110^. A higher quality of early hatchers could positively affect their development rate and decrease age at emergence with no trade-off between these two traits. The few studies that focused on maternal effects in damselflies reported weak or no impacts of the mother on her offspring quality^72,111,112^. In the current study we could not determine if maternal effects had an impact on life history and physiology traits, but it is worth investigating in the future.

We found ecologically important temperature effects on life history traits which did not show SMPEs. As expected, the high temperature decreased development time, and the shorter development time resulted in a lower mass at emergence. This elevated temperature-driven trade-off was somewhat reduced by increased growth rates at the high temperature, yet, the increase of growth rate was not strong enough to fully compensate the shorter development time. A similar incomplete compensating mechanism under warming was shown in previous studies, including studies on damselflies^113^ and is considered one major mechanism for the here observed temperature-size rule where animals get smaller at higher temperatures^114^.

It has been demonstrated that non-consumptive predator effects can change prey life history traits^32,115,116^, and could therefore potentially weaken or remove SMPE in prey, by, for example, reduced foraging rate in prey due to predator avoidance^117–119^. Here, we show that predator cues affected damselfly life history, but without having an effect on SMPEs. Specifically, predator cues reduced larval growth rate, leading to a lower mass at emergence, but only in the high temperature treatment (predator cue × temperature interaction for growth and mass). This suggests that the expected temperature rise will likely increase non-consumptive predator stress in *I. elegans*, with potentially negative fitness consequences. Similar results were shown in previous studies on other ectotherms^45,46^, including a damselfly^120^. These results could be explained as follows: predator stress increases physiological stress in prey, causing more energy to be allocated to costly defence mechanism rather than growth rate^121^. Taken together, current and previous results indicate that warming temperature may magnify the effects of predator-induced stress in prey, but that the increased predation stress may not affect SMPEs in prey.

The increased temperature lead to a weak SMPE in protein content (phenology × temperature interaction, *p* = 0.074), a fundamental component of various body structures, including muscles^122^, whereby the early larvae in the mixed group had a higher protein content under warming. This matched our second prediction of SMPEs being stronger or more likely at the high temperature. SMPE may be more likely under warming because a higher metabolism allows faster and more pronounced reactions to interactions between organisms, as well as the latter being stronger in general. In cannibalistic species, increased interactions result in higher cannibalism rates^7,123^. As conspecifics represent a rich source of proteins for cannibals^124^, increased cannibalism may lead to a higher protein content which can have positive effects on body condition during the larval stage^125,126^. As proteins make up an important part of the swim muscles in damselfly larvae, it may contribute to a better predator escape performance. Furthermore, this may generate positive carry-over effects across metamorphosis in the adult stage. For instance, proteins play an important role in ensuring proper wing elasticity, and as building blocks of flight muscles and the exoskeleton^127–129^. Intriguingly, the increased protein content under warming was not traded off against a faster growth rate, as it happened in body mass. This suggests that the larvae invest more energy into proteins than into other traits shaping final body size. It would be interesting to study in detail into which tissues the early hatched individuals invested more in the context of SMPEs.

We did not detect SMPEs in immune function (PO activity) and energy storage (fat content). These traits had similar values across all experimental treatments, suggesting fixed responses. These results are surprising because previous studies showed that PO activity and fat storage increased under warming, and decrease under predator pressure but, again, when larvae were reared individually^50,130^. That the physiological traits did not follow the SMPE hypothesis confirms previous results in *L. sponsa*^29^. Yet, in the latter species trait values showed plastic responses when individuals were exposed to time stressed conditions: PO activity decreased and fat content increased^29^. In the current experiment we did not impose time stress, but it would be interesting to study this stress on SMPE in *I. elegans* and link it with variable voltinism in this damselfly.

In summary, our results confirm that SMPEs caused by differences in hatching phenology are an important factor that by shaping survival and emergence success can promote early emergence of amphibious and cannibalistic organisms in a population. Other central findings of current study were that warming and non-consumptive effects imposed by a top predator did not affect SMPE for life history traits, yet warming did generate a weak SMPE for larval protein content that may adaptively carry over to the adult stage. In agreement with theory^20^, and current results, we suggest that given the high tendency for larval cannibalism, SMPEs in *I. elegans* could lead to directional selection for early adult breeding.

## Supplementary Information

Supplementary Information.

## Data Availability

All data generated or analyzed during this study are available in Zenodo repository (https://doi.org/10.5281/zenodo.6866384).
