# Supplementary material for: Warming and predation risk only weakly shape size-mediated priority effects in a cannibalistic damselfly
Source: Sci Rep. 2022 Oct 15;12:17324. doi: 10.1038/s41598-022-22110-6 (PMC9569353; doi:10.1038/s41598-022-22110-6)
Supplement: Supplementary file 1 — Supplementary Information. [file 41598_2022_22110_MOESM1_ESM.docx]

Supplementary information (SI)

**Warming and predation risk only weakly shape size-mediated priority effects in a cannibalistic damselfly**

Authors: Mateusz Raczyński^1^, Robby Stoks^2^, Szymon Sniegula^1^

Affiliations:

^1^ Department of Ecosystem Conservation, Institute of Nature Conservation, Polish Academy of Sciences, al. Adama Mickiewicza 33, 31-120 Krakow, Poland

^2^ Evolutionary Stress Ecology and Ecotoxicology, University of Leuven, Leuven, Belgium

Corresponding authors:

Mateusz Raczyński, raczynski@iop.krakow.pl

Szymon Sniegula, szymon.sniegula@gmail.com

APPENDIX 1


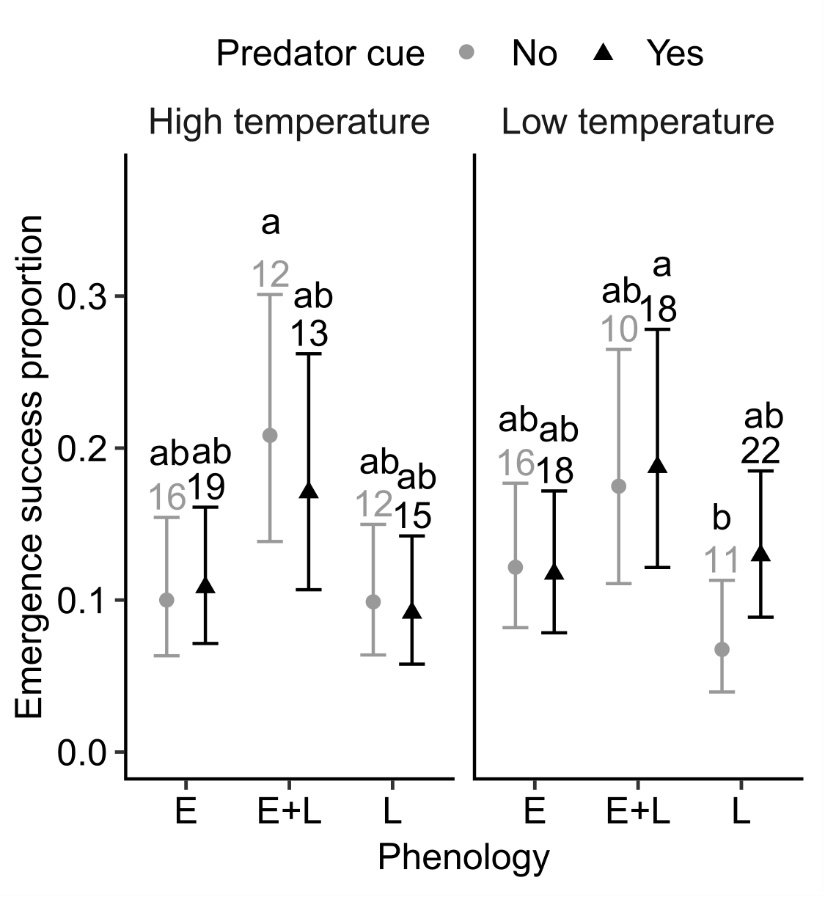


Fig S1 Emergence success across different phenology groups (E, E+L, L and L+E), temperatures (high and low) and predator cues (present and absent). Error bars indicate estimated 95% CI. The numbers over error bars represent the N count within each group. E – early larvae group, E+L – early larvae in mixed phenology group, L – late larvae group, L+E – late larvae in mixed phenology group. Because of low sample sizes in the L+E phenology group, the L+E group was removed from all analyses and plots.


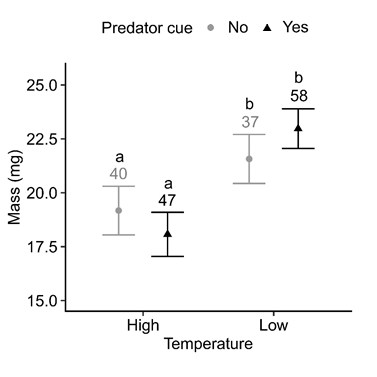


Fig S2 Mass at emergence across different temperatures (high and low) and predator cues (present and absent). Error bars indicate estimated 95% CI. The numbers over error bars represent the N count within each group. Letter codes indicate significant differences between temperature and predator cue groups.


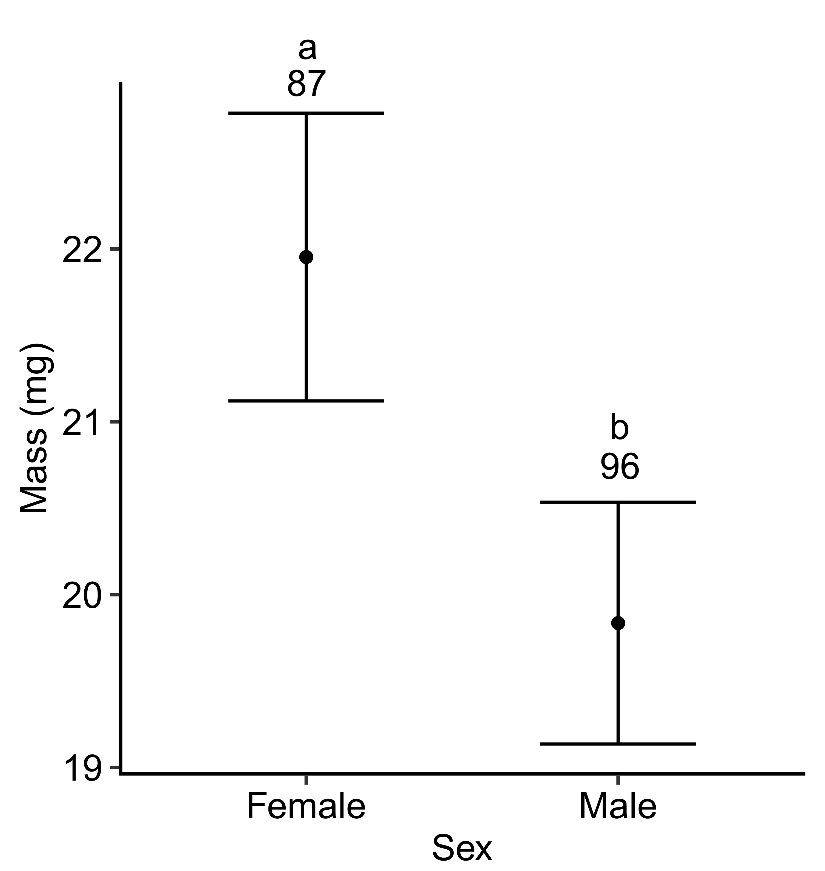


Fig. S3 Mass difference between sexes in *Ischnura elegans*. Error bars indicate estimated 95% CI. The numbers over error bars represent the N count within each group. Letter codes indicate significant differences between sexes.


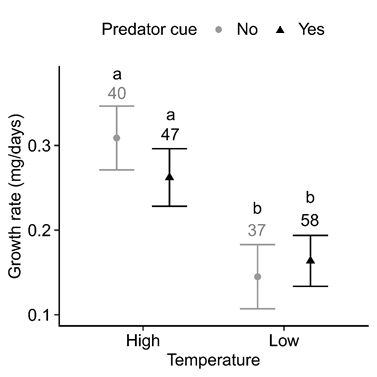


Fig S4 Growth rate across different temperatures (high and low) and predator cues (present and absent). Error bars indicate estimated 95% CI. The numbers over error bars represent the N count within each group. Letter codes indicate significant differences between temperature and predator cue groups.


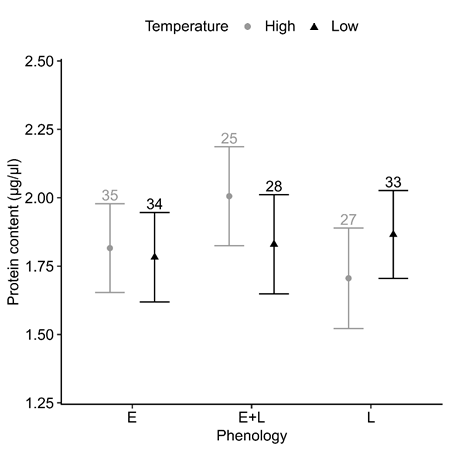


Fig S5 Protein content across different phenologies (E, E+L, and L) and temperatures (high and low). Error bars indicate estimated 95% CI. The numbers over error bars represent the N count within each group. Letter codes were not added due to lack of support of statistical significant differences between groups from post-hoc tests.


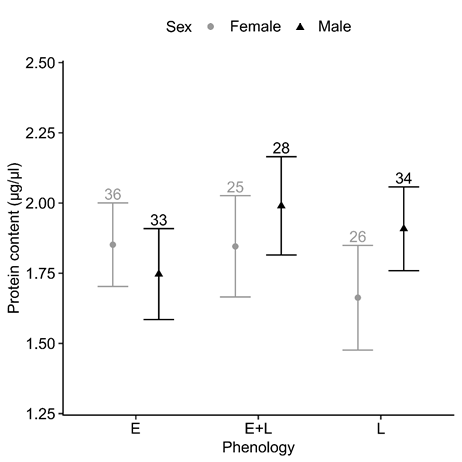


Fig S6 Protein content across different phenologies (E, E+L, and L) and sexes (male and female). Error bars indicate estimated 95% CI. The numbers over error bars represent the N count within each group. Letter codes were not added due to lack of support of statistical significant differences between groups from post-hoc tests.
